# Can uptake of childhood influenza immunisation through schools and GP practices be increased through behaviourally-informed invitation letters and reminders: two pragmatic randomized controlled trials

**DOI:** 10.1186/s12889-022-14439-4

**Published:** 2023-01-20

**Authors:** Rebecca Howell-Jones, Natalie Gold, Sarah Bowen, Amanda Bunten, Karen Tan, Ayoub Saei, Sarah Jones, Pauline MacDonald, Robin Watson, Kirsty F. Bennett, Tim Chadborn

**Affiliations:** 1 Public Health England Behavioural Insights, Wellington House, 133-155 Waterloo Road, London, SE1 8UG UK; 2 Behavioural Practice, KPUK, 4 Millbank, Westminster, London, SW1P 3JA UK; 3 Centre for Philosophy of Natural and Social Science, London School of Economics and Political Science, Houghton Street, London, WC2A 2AE UK; 4 School of Economics, Sir Clive Granger Building University Park, Nottingham, NG7 2RD UK; 5 UK Health Security Agency, Statistics, Modelling and Economics Department, 61 Colindale Ave, London, NW9 5EQ UK; 6 NHS England, Childhood Flu Immunisation Taskforce Programme Manager (Public Health Commissioning Central Team), London, UK; 7 NHS England, Programme Director, National Child Flu Immunisation Taskforce (Public Health Commissioning Central Team), London, UK; 8 Independent Nurse Consultant, Infection Matters Limited, London, UK; 9 Department of Anthropology, Durham University, Dawson Building, South Road, Durham, DH1 3LE UK; 10 Department of Psychology, University of Warwick, Coventry, UK; 11 Department of Behavioural Science and Health, Cancer Communication and Screening Group, University College London, London, UK

**Keywords:** Behaviourally-informed invitation letter, Childhood flu, Influenza immunization, Primary care, Text message reminders, UK childhood influenza immunisation programme, Vaccination

## Abstract

**Background:**

The UK is rolling out a national childhood influenza immunisation programme for children, delivered through primary care and schools. Behaviourally-informed letters and reminders have been successful at increasing uptake of other public health interventions. Therefore, we investigated the effects of a behaviourally-informed letter on uptake of the vaccine at GP practices, and of a letter and a reminder (SMS/ email) on uptake at schools.

**Methods and results:**

Study 1 was a cluster-randomised parallel trial of 21,786 two- and three-year olds in 250 GP practices, conducted during flu season (September to January inclusive) 2016/7. The intervention was a centrally-sent behaviourally-informed invitation letter, control was usual care. The proportion of two- and three-year olds in each practice who received a vaccination by 31st January 2017 was 23.4% in the control group compared to 37.1% in the intervention group (OR = 1.93; 95% CI = 1.82, 2.05, *p* <  0.001).

Study 2 was a 2 (behavioural letter vs standard letter) × 2 (reminder vs no reminder) factorial trial of 1108 primary schools which included 3010 school years 1–3. Letters were sent to parents from providers, and reminders sent to parents from the schools. In the standard-letter-no-reminder arm, an average of 61.6% of eligible children in each school year were vaccinated, compared to 61.9% in the behavioural-letter-no-reminder arm, 63.5% in the standard-letter-plus-reminder arm, and 62.9% in the behavioural-letter-plus reminder condition, *F*(3, 2990) = 2.68, *p* = 0.046. In a multi-level model, with demographic variables as fixed effects, the proportion of eligible students in the school year who were vaccinated increased with the reminder, *β* = 0.086 (0.041), *p* <  0.036, but there was no effect of the letter nor any interaction effect.

**Conclusion:**

Sending a behaviourally informed invitation letter can increase uptake of childhood influenza vaccines at the GP surgery compared to usual practice. A reminder SMS or email can increase uptake of the influenza vaccine in schools, but the effect size was minimal.

**Trial registration:**

Study 1: Trial registration: ClinicalTrials.gov Identifier: NCT02921633.

Study 2: Trial registration: ClinicalTrials.gov Identifier: NCT02883972.

**Supplementary Information:**

The online version contains supplementary material available at 10.1186/s12889-022-14439-4.

## Introduction

In 2012, the UK Joint Committee on Vaccination and Immunisation recommended a national childhood influenza immunisation programme be rolled out to 2- to 16-year olds, using a live attenuated influenza vaccine [1]. Roll out of the programme began in 2013/14 with 2- and 3-year olds offered the vaccine through general practice and seven pilot areas offering the vaccine to primary school-aged children mostly in the school setting. By 2016/17 the programme had expanded to include 2- to 6-year olds nationally. In pilot areas the vaccine continued to be offered to all primary school aged children.

In 2015/16 nationally, uptake in school years 1 and 2 was 53.6% [2]. In the pilot areas offering the vaccine to all primary school children (5–11 years), the overall uptake of vaccination in 5–11 years olds was 57.9% in 2015/6, which was a lower uptake than the 60.4% achieved in the previous year, 2014/5. The pilot areas also had lower uptake as age increased, with 62.6% in Year 1 and 54.7% in Year 6 in 2015/16 (see also [3]). Delivery methods had considerable impact on uptake, with higher uptake in those areas that delivered flu immunisation through schools (55.6%) than General Practices (GPs) (32.9%) and/or pharmacies (16.1%). There may be higher uptake in schools because parents feel the setting is an advantage, since it avoids the inconvenience of having to take their child to the GP, as well as there being beneficial effects of children being vaccinated in the company of their classmates [4–6]. Analysis has also shown that uptake is associated with deprivation and ethnicity. Children in the poorest deprivation quintile have been shown to be 19% less likely to receive influenza vaccine than those in the wealthiest quintile [3], and areas that have a higher percentage of black and ethnic minority (BAME) population have been shown to have a lower uptake: 14.5% lower uptake in those areas in the highest quartile (> 34% BAME) compared to the lowest quartile (< 5% BAME) [7].

National template letters are published on the Annual Flu Programme website,^1^ for general practices and immunisation providers for schools to use. The letter for immunisation that is posted to parents/guardians from general practice asks them to book an appointment and to take their child to the practice to receive the immunisation. The letter for immunisation from schools asks parents/guardians to complete and return an enclosed consent form. While uptake in schools is higher than in general practice or pharmacies, there remains approximately 30% of children for whom consent is neither given nor actively withheld [8].

Much of the literature on vaccine uptake focusses on vaccine hesitancy and on knowledge, attitudes and beliefs about vaccines (e.g. [4, 9–12]). However, barriers to childhood vaccinations also include access issues, such as parents who might want their child to be vaccinated but experience difficulties in gaining an appointment [13]. People may intend well, but intentions do not always translate into action [14]. There is a role for interventions that change health behaviours by targeting environmental stimuli, in order to move them to action [15].

Applying behavioural science to invitation letters can increase the uptake of health interventions. For example, a 4% increase in the uptake of the NHS Health Checks was achieved by simplifying the invitation letter and adding a tear-off slip where individuals could note the time and date of their first appointment, as a memory aid [16]. Behaviourally-informed letters have also been effective at getting parents to take action on behalf of their children: an enhanced National Child Measurement Programme (NCMP) letter sent to parents of children with a higher than normal body mass index (BMI) led to them being more likely to enroll their children in weight management services [17].

Reminders can also increase uptake of interventions, with text message reminders having been successful at increasing uptake of NHS Health Checks [18] and breast cancer screening [19]. A randomised controlled trial (RCT) that sent text-message reminders for the influenza vaccination to at-risk adults under 65 years reported a 2.6% increase in uptake, but this was not statistically significant [20]. Both telephone and text message reminders have been shown to increase attendance at hospital appointments [21–23].

In 2016/17 Public Health England Behavioural Insights (PHEBI) worked with NHS England and Public Health England (PHE) immunisation team to redesign the template invitation letters for general practice and primary schools. In this paper we report on two RCTs, the first clustered at the GP level, and the second at school level, to test whether behaviourally-informed letters can increase uptake of the childhood influenza (flu) vaccination. In both cases, clustering was a pragmatic choice to deliver the intervention and reduce the potential for cross-treatment contamination.

## Study 1: cluster-randomized controlled parallel trial of the effect of a behaviourally-informed letter on childhood flu vaccination uptake in general practice

### Aim

To test whether a behaviourally-informed invitation letter will increase uptake of childhood flu vaccine in general practice (compared to standard practice).

### Methods

The trial was registered at ClinicalTrials.gov on 03/10/2016 at NCT02921633. This was a cluster randomised controlled parallel trial using stratified randomisation. Participants were children aged 2 and 3 years on 31st August 2016 registered at 257 GP practices in seven Clinical Commissioning Groups (CCGs) in the area covered by the Immunisation and Screening Team of PHE East of England / NHS England Midlands & East (NHS Mid Essex, NHS North East Essex, NHS Thurrock, NHS West Essex, NHS Basildon and Brentwood, NHS Castlepoint and Rochford and NHS Southend CCG). CCGs are groups of general practices (GPs) which come together in each area to commission the best services for their patients and population. GP practices were the unit of randomisation.

#### Interventions

Parents of eligible children at practices in the intervention arm were sent the behaviourally-informed invitation letter by post with minimal local adjustments (e.g. reflecting the sender and flu clinic booking arrangements). A number of behavioural techniques were used, several of which were coded according to the Behavioural Change Technique (BCT) Taxonomy [24]. The letter included the GP practice name, as the NHS is a trusted and credible source when it comes to health issues (BCT 9.1: Credible source). The letter was reduced in length, simplified and made more salient by highlighting key points or actions required, thereby reducing the cognitive effort required to process the information and identify the action required [25–28]. It was personalised with the name of the child and the parent, increasing the letters personal relevance, and had a tear-off slip where individuals could note the time and date of their appointment, addressing implementation intentions (BCT 1.4: Action planning and goal achievement [29]).

Other behavioural techniques that were used included framing the message as a gain (*‘This vaccination programme is in place to help protect your child against flu’* - BCT 13.2: Framing), providing information about the health consequences of flu and salience of consequences (*‘Flu can be an unpleasant illness and sometimes causes serious complications’* - BCT 5:1: Information about health consequences and 5.2: Salience of consequences), providing information about social and environmental consequences [24] (*‘Vaccinating your child will also help protect more vulnerable friends and family by preventing the spread of flu’* - BCT 5.3: Information about social and environmental consequences) and framing and reducing friction by eliminating the barrier of cost (*‘The vaccination is free and recommended for young children … ’* - BCT 13.2: Framing). A full list of the behavioural techniques utilised and their rationales can be found in Table 1. A copy of the letter can be found in Supplementary Material 1.

**Table 1:** Behavioural-science informed changes to the flu invitation letter and their rationale

Formatting and phrasing used in new template invitation letter	Behavioural rationale
**Formatting**	
Reduced in length and made simpler.	*Simplification:* Reduces the cognitive effort required to process the information and identify the action required [25].
Formatted text to highlight the key points of note/ actions required.	*Salience:* Reduces cognitive effort to identify action required [26–28].
GP practice named	*Messenger effect:* NHS is a trusted brand when it comes to medical/ health issues. (BCT 9.1 Credible Source [24])
Tear-off appointment time slip	*Action Planning:* Addressing implementation intentions by providing a tear off slip to record the date time and location of the appointment [29]. Parent actively commits to implement intention as they write down the date, time and location of the appointment. (BCT 1.4 Action Planning [24])
**Phrasing**	
Dear [Name]	*Personalisation:* Increases personal relevance and focuses attention.
«Insert child’s first name»‘s annual flu vaccination is now due	*Personalisation and personal salience:* Using Child’s name increases personal relevance and stating ‘due’ creates a sense of urgency.
This vaccination programme is in place to help protect your child against flu.	*Gain-framed message:* evidence indicates gain-framed messages may be more effective than loss-framed for preventative behaviours (BCT 13.2 Framing [24])
Flu can be an unpleasant illness and sometimes causes serious complications.	*Information about Health Consequences* and *Salience of Consequences:* highlighting the negative effects of flu (BCT 5.1 Information about Health Consequences and 5.2 Salience of consequences [24])
Vaccinating your child will also help protect more vulnerable friends and family by preventing the spread of flu.	*Providing information about social and environmental consequences: making it personally relevant. Framing/Reframing:* framing the child’s vaccination in terms of protecting other loved ones in order to change emotions about performing the behaviour (BCT 5.3 Information about social and environmental consequences, 13.2 Framing [24])
Please phone [insert number] to book an appointment for [insert child’s name]‘s flu vaccination.	*Action orientated:* providing clear action focused instruction including telephone number to make the behaviour easy to perform and providing child’s name to increase personal salience (BCT 4.1 Instruction on how to perform a behaviour [24])
The vaccination is free and recommended for young children …	*Framing/ Reducing friction:* to perform the behaviour: Reduces friction costs by eliminating the barrier of cost since there is anecdotal evidence that parents do not realise it is free or annual. Note: the evidence on use of ‘free’ is mixed as this can be deemed as less valuable, particularly by some cultures. (BCT 13.2 Framing, 6.3 Information about others’ approval [24])
… and will be given by a quick and simple spray up the nose	*Framing effect:* Encouraging uptake of this method because it is appealing and not painful (compared to traditional ideas of a vaccination being an invasive injection) (BCT 13.2 Framing [24])

The letters were sent centrally by the Child Health Information System (CHIS) team on behalf of the practice between 21 and 25 October 2016.

Parents of eligible children registered at general practices in the control arm were only provided with the usual communication from their practice. GPs are contracted to call and recall for immunizations. The National Institute of Health and Care Excellence (NICE) recommends contacting people for immunisations by a variety of methods, including text messages, letters, emails, phone calls from staff or an auto dialler, social media, or a combination of methods, as well as informing and inviting people during face-to-face interactions whenever the opportunity arises [30].

We surveyed the GP practices to obtain information on their invitation communication with parents; 25% (63 out of 257) practices responded to the survey. The majority of practices confirmed that they had invited all eligible patients (76%), 17% to some parents and only one practice to no parents. Whilst the majority of practices reported sending letters to some or all parents, practices also used phone calls, text messages and opportunistic occasions. Recall was however less frequently undertaken by practices.

#### Outcomes

The pre-registered primary outcome for this study was immunisation uptake by eligible children (i.e. those aged 2 and 3 years on 31st August 2016) at primary care. Anonymised individual-level data was extracted from the CHIS on 28th March 2017. This included flu vaccine history in 2016/17 and 2015/16 flu seasons, age, gender, and GP practice.

#### Sample size

The sample was fixed as all practices in the participating areas. Power calculations indicated that, with 120 general practices, we would have 90% power to detect a 5% absolute increase in uptake, from 34 to 39% in children aged 2–3 years.

#### Randomisation

A stratified randomisation strategy was used. In total 14 strata were created (all of the possible combinations of the seven CCGs and whether or not they were SystmOne users: SystmOne is a software system used in some practices; the databases of practices with SystmOne were automatically updated with a copy of the letter sent on the patient’s record and any flu immunisations recorded on the GP practice system were automatically transferred to CHIS, whereas other practices may have done this manually). Randomisation was by general practice. General practices within each strata were randomly assigned to the intervention group (Group 1) or control group (Group 2). A code was developed in Stata by researchers at PHE to randomise practices. General practices in the intervention group were informed that CHIS was sending an invitation letter to the parents of two- and three-year olds as part of a trial to try to increase immunisation uptake. Parents were not informed that the letters were being sent as part of a trial. General practices in the control group were not informed about the trial.

#### Statistical methods

Classical linear logistic regression models were run, with a binary outcome variable equal to 1 if an eligible child was vaccinated, and 0 otherwise. The models included previous vaccination and demographic variables (age and sex) as fixed effects, in addition to the intervention. It also included GP practice as a random effect, to account for variation not explained by the fixed effects. Analyses were carried out using R, version 4.0.0.

### Results

**Fig. 1:**
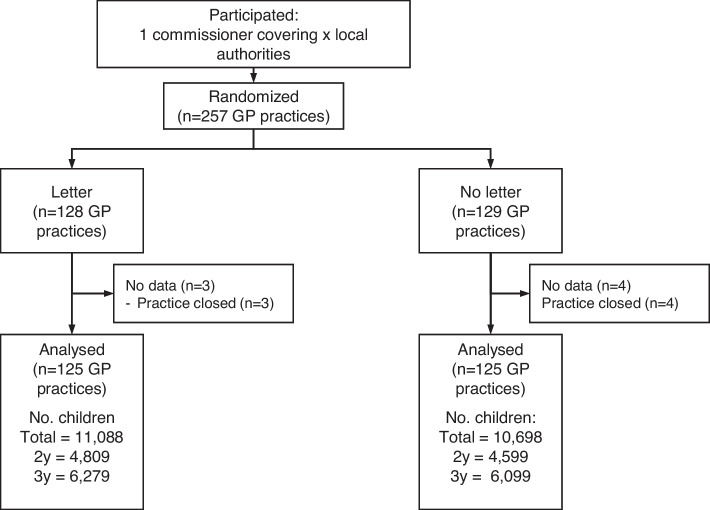
GP Flu participant flow diagram

In total, 257 GP practices were randomised into the two conditions; however, 7 practices were subsequently dropped because they closed. This left 21,786 patients across 250 GP practices who were included in the analysis. Across the practices, there was a median of 73 patients with an interquartile range of 75. There were 125 GP practices with 10,698 eligible patients in the control condition and 125 practices with 11,088 eligible patients in the intervention. Figure 1 shows the participant flow diagram for this study.

The demographics recorded in this study were not significantly different between the intervention and the control group (see Table 2.)

**Table 2:** Counts and percentages showing breakdown of demographic variables across groups and chi-square tests of independence

		Control *n* = 10698	Intervention *n* = 11088	χ^2^ (df)	p
Sex [n (%)]	Male	5446 (50.9%)	5687 (51.3%)	0.304 (1)	0.58
	Female	5252 (49.1%)	5401 (48.7%)		
Age [n (%)]	2-year-old	4599 (43.0%)	4809 (43.4%)	0.308 (1)	0.579
	3-year-old	6099 (57.0%)	6279 (56.60%)		
Previous Vaccine [n (%)]	Previously had vaccine No previous vaccine	261 (2.44%) 10437 (97.56%)	257 (2.32%) 10831 (97.68%)	0.298 (1)	0.58

The proportion of children who were vaccinated was higher in the intervention group, who were sent the behavioural insights letter (37.1%, *n* = 4113) than the control group, who were not sent the letter (23.4%, *n* = 2499), OR = 1.93; 95% CI = 1.82, 2.05, *p* <  0.001. This represents a 13.7% change in absolute terms and a 48.8% increase in relative terms.

Model 1 showed that after controlling for demographic variables, the intervention group were significantly more likely to be vaccinated than the control group (OR = 2.24; 95% CI = 1.67, 3.00, *p* <  0.001). This conclusion remained unchanged after adding interaction effects in Model 2 (OR = 2.02; 95% CI = 1.49, 2.75, *p* <  0.001). Although there were statistically significant differences in vaccination rates across GP practices and across CCGs, there is no evidence to reject the hypothesis that there was an equal effect of general practice variation across intervention and control groups, since there were no significant differences between the slopes for intervention across general practices (χ^2^ (2) =0.09, *p* = 0.96) or across CCGs (χ^2^ (2) =4.18, *p* = 0.12). The results of the models can be found in Table 3.

**Table 3:** Logistic regression models predicting the odds of being vaccinated

	Model 1: (Pseudo R^2^ = 0.15)		Model 2: (Pseudo R^2^ = 0.41)	
	Odds Ratio (95% CI)	*p*	Odds Ratio (95% CI)	*p*
Intercept	0.05 (0.03, 0.09)	< 0.001	0.06 (0.04, 0.09)	< 0.001
Intervention	2.24 (1.67, 3.00)	< 0.001	2.02 (1.49, 2.75)	< 0.001
Female (reference category male)	1.04 (0.98, 1.11)	0.223	0.96 (0.87, 1.05)	0.371
3-year old (reference category 2-year old)	0.94 (0.88, 1.01)	0.076	0.91 (0.83, 1.01)	0.067
Previously vaccinated (reference category not previously vaccinated)	3.96 (3.23, 4.85)	< 0.001	4.33 (3.28, 5.72)	< 0.001
SystmOne	5.77 (3.94, 8.45)	< 0.001	5.77 (3.94, 8.45)	< 0.001
Intervention *Female	–	–	1.16 (1.02, 1.32)	0.024
Intervention*3-year old	–	–	1.06 (0.93, 1.21)	0.362
Intervention* Previously vaccinated	–	–	0.83 (0.55, 1.24)	0.362
Variation between practices (SD)^1^	1.10	< 0.001	1.10	< 0.001
Variation between CCG (SD) ^2^	0.42	< 0.001	0.42	< 0.001
ICC	0.297		0.297	

^1^ The p value displayed by practice is from a log likelihood test between a model that included and omitted the random effect

^2^ The *p* value displayed by CCG is from a log likelihood test between a model that included and omitted the random effect

There were no significant differences between males and females in either model (Model 2: OR = 0.96; 95% CI = 0.87, 1.05, *p* = 0.37). There was a statistically significant interaction between gender and receiving the intervention (Model 2: OR = 1.16; 95% CI = 1.02, 1.32 *p* = 0.024). Children aged 3 years were slightly less likely to receive a vaccine but this was not significant (Model 1: OR = 0.94; 95% CI = 0.88, 1.01, *p* = 0.08). There was no statistically significant interaction between age and receiving the intervention (OR = 1.06; 95% CI = 0.93, 1.21, *p* = 0.36).

Children who had previously been vaccinated were more likely to be vaccinated (these could only have been 3-year olds, since 2-year olds were not eligible the previous year) and the effect remained significant after adjusting for variation in general practices (OR = 4.33; 95% CI = 3.28, 5.72, *p* <  0.001). There was no evidence of a statistically significant interaction between previous vaccination and receiving the intervention (OR = 0.83; 95% CI = 0.55, 1.24, *p* = 0.36).

Practices who used SystmOne reported higher uptake, (Model 2: OR = 5.77; 95% CI = 3.94, 8.45, *p* <  0.001).

## Study 2: randomized controlled trial of the effect of a behaviourally-informed letter and a reminder on childhood flu vaccination uptake schools (factorial design)

### Aim

The aim of this study was to determine whether applying behaviourally-informed interventions to the invitation process will improve uptake of childhood flu immunisation in school-based programmes.

Our specific hypotheses were:

1. Behaviourally-informed changes to the invitation letter will increase uptake of childhood flu vaccine in schools.
2. A reminder to return the consent form will increase uptake of childhood flu vaccine through schools

By using a factorial design, we were also able to investigate any interactions between the behaviourally-informed letter and the reminder.

### Methods

The trial was registered at ClinicalTrials.gov on 30/08/2016 at NCT02883972. This was an RCT with a 2 (behavioural letter vs. standard letter) × 2 (reminder vs no reminder) factorial design. Five of the six providers of childhood flu school vaccination in the Wessex, Leicester and Essex NHS England area teams agreed to take part (Solent NHS Trust, Dorset Healthcare University NHS Foundation Trust, Leicestershire Partnership NHS Trust, Boots UK and South Essex Partnership University NHS Foundation Trust). All primary schools in participating areas were eligible for inclusion. Each school vaccination session took place during a school day in the Autumn term. In the UK, parents are not required to be present for childhood vaccinations. The exact time and date of the sessions were chosen on a school-by-school basis. The trial took place from October to December 2016 during the delivery of the winter 2016/7 school-based flu vaccination programme.

#### Interventions

##### Intervention 1: Behaviourally-informed letter

A behaviourally-informed letter was sent via post by providers of childhood flu school vaccination to parents at those schools randomised to Intervention 1, with minimal local adjustments. The behavioural techniques used were the same as the ones used in the general practice trial except there was no personalisation (this was not feasible, since exactly the same letter is sent to all parents) or a tear-off appointment time slip (because the required action was returning a consent form, not booking an appointment). The schools letter also included a positive social norm statement (*Last year, most children offered the vaccine in school had the immunisation’* – BCT 6.2: Social comparison). The formatting and phrasing, and the associated rationale for them are summarised in Table 4. A copy of the letter can be found in Supplementary Material 2. In all groups, schools gave out letters and an information leaflet, with information such as the possible side-effects of the vaccine, to children to take home to their parents.

**Table 4:** Behavioural insights informed changes to the flu invitation letter and rationale

Changes to letter	Behavioural rationale
**Formatting**	
Reduced in length and made simpler.	*Simplification:* Reduces the cognitive effort required to process the information and identify the action required [25].
We have formatted text to highlight the key points of note/ actions required.	*Salience:* Reduces cognitive effort to identify action required [26–28].
Provider letterhead plus NHS logo	*Messenger effect:* NHS is a trusted brand when it comes to medical/ health issues. (BCT 9.1 Credible Source [24])
**Phrasing**	
Your child’s annual flu vaccination is now due	Stating ‘due’ creates a sense of urgency.
This vaccination programme is in place to help protect your child against flu.	*Gain-framed message:* evidence indicates gain-framed messages may be more effective than loss-framed for preventative behaviours (BCT 13.2 Framing [24])
Flu can be an unpleasant illness and sometimes causes serious complications.	*Information about Health Consequences* and *Salience of Consequences:* highlighting the negative effects of flu (BCT 5.1 Information about Health Consequences and 5.2 Salience of consequences [24])
Please complete the enclosed consent form (one for each child) and return to the school [by/ within] [INSERT DATE or TIME FRAME]	*Action orientated:* providing clear action focused instruction including telephone number to make the behaviour easy to perform and providing child’s name to increase personal salience (BCT 4.1 Instruction on how to perform a behaviour [24])
The vaccination is free …	*Framing/ Reducing friction:* to perform the behaviour: Reduces friction costs by eliminating the barrier of cost since there is anecdotal evidence that parents do not realise it is free or annual. Note: the evidence on use of ‘free’ is mixed as this can be deemed as less valuable, particularly by some cultures. (BCT 13.2 Framing, 6.3 Information about others’ approval [24])
… and will be given by a quick and simple spray up the nose	*Framing effect:* Encouraging uptake of this method because it is appealing and not painful (compared to traditional ideas of a vaccination being an invasive injection) (BCT 13.2 Framing [24])
Last year, most children offered the vaccine in schools had the immunisation.	Social norms (BCT 6.2 Social comparison [24])

Parents at schools randomised to a control letter group were given usual practice, a standard letter invitation letter sent to all parents from the provider. They were sent the letter used by the immunisation provider during the previous year (with necessary updates). These letters varied from area to area.

##### Intervention 2: reminder

Schools randomised to a reminder group were asked by the local immunisation team to send a timely reminder message to parents through their usual email or SMS system. The reminders were sent 2 days before the deadline for returning the consent form for pragmatic reasons of implementation. The reminder messages were informed by behavioural insights and were action-orientated with a focus on scarcity and anticipated regret in both messages (e.g., “*ensure your child doesn’t miss out”* and “*There will be only one vaccination session at the school”*). The SMS and email reminder messages are shown in full in Table 5. Immunisation teams were given a standard operating procedure detailing how to make this request to schools. All schools had either email or SMS systems to communicate with parents.

**Table 5:** Text for SMS and email reminder messages

Reminder format	Message
SMS (160 characters limit)	Thank you for returning your child’s flu vaccine consent form. If you have not, please return it by xxxxxx to ensure your child doesn’t miss out.
Email	Subject: Flu vaccine consent form due xx/xx/xx: make sure your child doesn’t miss out Dear Parent/Guardian, Please return your child’s flu vaccine consent form by [*insert day and date*] to make sure your child doesn’t miss out. Thank you to those parents who have already done so. There will be only one vaccination session at the school. Last year, most children offered the vaccine in schools had the immunisation. [*The consent form is attached for use if you have lost the letter*]^a^ If you decide you do not want to vaccinate your child against flu, please return the consent form giving the reason. This will help us plan and improve the service Yours sincerely, SIGNED BY PROVIDER

^a^ This sentence was requested to be included only where it was feasible to include the original letter as an attachment

Schools in the control group for the reminder message were not specifically asked to send a reminder, so they did whatever would be usual practice, but we would not expect this to include a reminder.

We endeavoured to collect data on the implementation of the trial to understand how rigidly the protocols were being applied locally and what usual practice was. This unfortunately proved difficult and data could not be obtained. Nonetheless, we assessed whether providing schools with recommended text for reminder messages, and the request that they send reminders increased uptake.

#### Outcomes

The pre-registered primary outcome for this study was the proportion of eligible children in the school years for each of years 1–3 (i.e. those aged 5 to 7 years on 31st August 2016) who received influenza immunisation as part of the national childhood immunisation programme. We focused only on children in years 1–3 during the 2016/17 winter season as they were the target age group for the national childhood influenza immunization campaign. The secondary outcomes also pre-registered were the proportion for whom the consent form was returned, the proportion of consent forms returned with consent granted, and the proportion of consent forms returned with consent denied.

#### Data collection

Immunisation programme data were collected by providers of childhood flu school vaccination on a PHE template spreadsheet. Providers submitted school-level data throughout the 2016/17 flu season and the data used was submitted as part of usual practice. No individual-level data or personal identifiable information were collected by the study team.

Data on characteristics of the schools were obtained from the publicly available ‘Compare school and college performance’ UK government website.^2^ This included information on the proportion of children eligible for free school meals, the religious denomination of the school, and the type of school (state-funded primary, state-funded secondary, or independent). The postcode of schools was linked to Index of Multiple Deprivation (IMD) data using http://imd-by-postcode.opendatacommunities.org/ to obtain a quintile ranking for the school location. The top 20% were coded as deprived.

#### Sample size

The sample was fixed by the number of schools in participating areas. Power calculations indicated that with the original number of schools available (~ 1700 schools and six providers) and assuming no variation between local authorities and an average of 100 children in years 1–3 in each school, we would have 90% power to detect a 1% absolute increase in uptake, from 63 to 64%.

#### Randomisation

A stratified method of randomisation was used with each local authority representing a separate strata. Randomisation was at school level. The intervention was a 2 × 2 factorial design, with schools within each strata randomly assigned to one of four interventions arms, one for each combination of interventions. For every school, a random number between − 1 and 1 was drawn from a uniform distribution. The schools were ordered according to the random number that had been generated for them; then the lowest 25% were assigned to Arm 1, the next 25% Arm 2 and so on. This was done separately for each strata. We did not know at the time of randomisation which schools had email/SMS systems, so we did not include this in the randomisation.

Providers knew which school was in each arm, but neither the schools nor the parents who received the interventions knew that they were in a trial.

#### Statistical methods

We used multi-level Generalised Linear Model (GLM) regression models to predict the proportion of eligible children in the school year receiving a vaccine, with school year and school as random effects, and demographic variables as fixed effects. As random effects are included in the model there is no need to cluster standard errors. Analyses were carried out using Stata, Version 15.

### Results

**Fig. 2:**
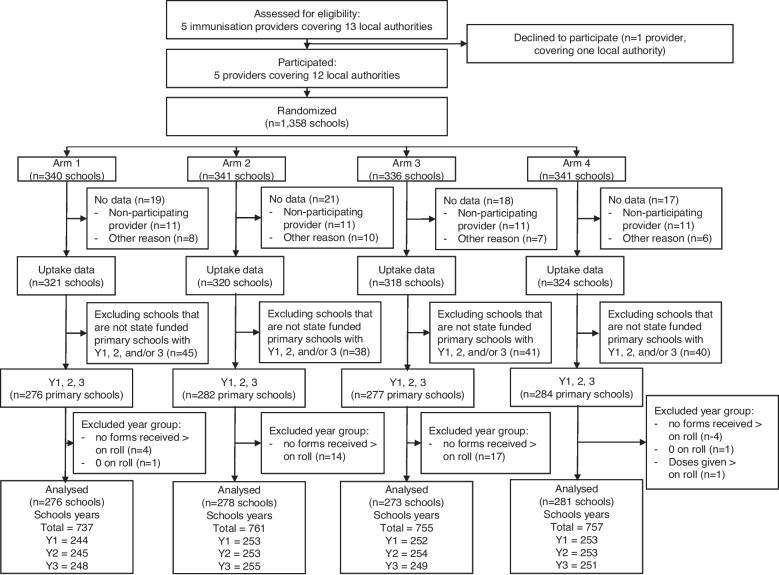
School Flu participant flow diagram

At randomisation there were 1358 schools and five providers in the trial. After excluding schools for which data were not available (one provider decided not to participate after randomisation.*n* = 44 and other reasons such as the school having closed), there were 1283 schools remaining. Only school years 1, 2 or 3 were included in the analysis (schools labelled as state-funded secondary schools (*n* = 6: 0.5%) were excluded as there were no state-funded secondary schools in the control or letter condition groups). Data for some of our independent variables (student ethnicity and free school meal eligibility) was not available for independent schools. Therefore, we excluded the independent schools from the analysis. We further excluded those school-years where the number of forms received or doses given was greater than the number of pupils on roll and where no children were recorded as being on roll. Fig. 2 shows the participant flow diagram for this study.

After exclusions, there were four providers with 1108 schools and 3010 school years in the analysis. For 892 (80.4%) of schools we observed 3 school years of childhood vaccination behaviour (Year 1, 2, and 3). Across the school years, there was a median of 42 children eligible for a vaccination, with an interquartile range of 34 (from 26 to 60).

Some of the demographic variables were not distributed evenly across groups (see Table 6). These were the percentage eligible for free school meals *F*(3, 2990) = 2.88, *p* = 0.035; the religious denomination of the school χ^2^ (15) = 57.41, *p* <  0.001; and whether the school was in an urban or a rural location, χ^2^ (3) = 13.226, *p* = 0.004.

**Table 6:** Outcome measures and breakdown of demographic variables across experimental conditions^a^

	Treatment Group				Overall	χ^**2**^/F (df) ^b^	p
	Control	Letter	Reminder	Letter & Reminder			
**% Children vaccinated in the school year (mean (SD))**	61.57 (15.61)	61.94 (15.60)	63.53 (14.13)	62.87 (14.04)	62.47 (14.88)	2.68 (32990)	0.046
**% Consent forms returned**	73.55 (15.85)	75.67 (15.43)	76.18 (14.72)	75.97 (14.19)	75.34 (15.1)	4.84 (3, 2990)	0.002
**% Consent given**	64.92 (15.07)	65.46 (15.58)	66.96 (13.89)	66.32 (13.55)	65.91 (14.57)	2.91 (3, 2990)	0.033
**% Consent refused**	8.63 (7.33)	10.21 (8.70)	9.22 (8.13)	9.65 (7.71)	9.43 (8.00)	5.23 (32990)	0.001
**Number of flu vaccine doses given in the school year (mean (SD))**	27.75 (16.71)	27.98 (17.90)	27.95 (16.48)	28.21 (18.04)	27.97 (17.28)	0.09 (3, 2990)	0.967
**Children eligible for flu vaccine in the school year (mean (SD))**	45.86 (26.09)	45.22 (26.24)	45.22 (26.24)	45.41 (28.13)	45.59 (27.05)	0.11 (3, 2990)	0.953
**Number of consent given**	29.26 (17.28)	29.46 (18.66)	29.55 (17.26)	29.67 (18.78)	29.49 (18)	0.07 (3, 2990)	0.976
**Number of consent refused**	3.79 (3.68)	4.66 (4.84)	3.94 (3.70)	4.19 (3.86)	4.14 (4.06)	6.64 (3, 2990)	0.000
**Number of consent returned**	33.05 (19.17)	34.12 (21.45)	33.49 (19.33)	33.86 (21.14)	33.63 (20.29)	0.39 (3, 2990)	0.759
**% Eligible for free school meals in the school (mean (SD))**	12.18 (9.74)	11.18 (9.13)	12.48 (9.93)	12.33 (9.17)	12.04 (9.51)	2.88 (32990)	0.035
**Average ethnic composition of school**							
% White	85.42	83.87	84. 18	84.69	84.54	0.98 (3, 2990)	0.402
% Mixed	4.85	4.92	4.68	4.54	4.75	2.28 (3, 2990)	0.077
% Asian	4.85	6.59	6.49	6.02	5.99	2.32 (3, 2990)	0.073
% Black	3.01	2.92	2.86	2.83	2.84	0.14 (3, 2990)	0.939
% Other	1.13	1.14	1.23	1.12	1.16	0.57 (3, 2990)	0.632
**Religious denomination of the school (n (%))**						57.41 (15)	< 0.001
Catholic	56 (7.46)	52 (6.87)	77 (10.24)	24 (3.27)	209 (6.98)		
Church of England	229 (30.49)	222 (29.33)	205 (27.26)	239 (32.56)	895 (29.89)		
Other Christian Faith	0 (0)	0 (0)	0 (0)	3 (0.41)	3 (0.10)		
Sikh	0 (0)	0 (0)	3 (0.40)	0 (0)	3 (0.10)		
Other	0 (0)	0 (0)	3 (0.40)	0 (0)	3 (0.10)		
No religion	466 (62.05)	483 (63.80)	464 (61.70)	468 (63.76)	1881 (62.83)		
**Deprivation (n (%))**						3.880 (3)	0.275
High	83 (11.05)	88 (11.62)	102 (13.56)	77 (10.49)	350 (11.69)		
Low	668 (88.95)	669 (88.46)	650 (86.44)	657 (89.51)	2644 (88.31)		
**Urban/rural area (n (%))**						13.226 (3)	0.004
Urban	512 (68.18)	497 (65.65)	525 (69.81)	451 (61.44)	1985 (66.30)		
Rural	239 (31.82)	260 (34.35)	227 (30.19)	283 (38.56)	1009 (33.70)		
**School Year (n (%))**						0.173 (6)	1.000
1	252 (33.55)	251 (33.16)	251 (33.38)	243 (33.11)	997 (33.30)		
2	251 (33.42)	252 (33.29)	254 (33.78)	244 (33.24)	1001 (33.43)		
3	248 (33.02)	254 (33.55)	247 (32.85)	247 (33.65)	996 (33.27)		

^a^For 16 schools in the trial, an inconsistency in the data for number of consent forms returned led us to drop these observations from analysis

of return of consent forms, though they were included in the analysis of the primary outcome measure (uptake)

^b^Chi square/ one-way ANOVA tests, as appropriate

The proportion of eligible children in the school year who received a vaccination differed across groups, *F*(3, 3010) = 2.62, *p* = 0.049: In the control group, an average of 61.6% of eligible children in each school year were vaccinated, compared to 61.9% in the letter group, 63.5% in the reminder group, and 62.9% in the letter and reminder group (see Table 6). When a multi-level model was run that included school and school year nested within school as random effects, which adjusted for demographic variables, there was a statistically significant increase in vaccination rates due to the reminder *β* = 0.086, *p* = 0.036. However, there was no effect of the letter, *β* = 0.029, *p* = 0.476, or the interaction of letter and reminder *β* = − 0.036, *p* = 0.533. The full model is shown as Model 1 in Table 7.

**Table 7:** Multi-level GLM models predicting the outcomes, school and school year nested in schools as random effects*

	Model 1: Proportion of flu vaccine doses given (number given of number of vaccine doses possible)		Model 2: Number of consent forms returned		Model 3: Number of consent forms where consent was granted		Model 4: Number of consent forms where consent was refused	
	***β*** (SE)	***p***	***β*** (SE)	***p***	***β*** (SE)	***p***	***β*** (SE)	***p***
**Treatment (base = control)**								
**Letter**	0.029 (0.041)	0.476	0.129 (0.059)	0.03	0.032 (0.040)	0.42	0.174 (0.058)	< 0.001
**Reminder**	0.086 (0.041)	0.036	0.150 (0.059)	0.01	0.100 (0.040)	0.01	0.057 (0.059)	0.33
**Letter**^a^**Reminder**	−0.036 (0.058)	0.533	−0.118 (0.084)	0.16	− 0.052 (0.057)	0.37	−0.068 (0.083)	0.41
**Free school meals (%)**	−0.017 (0.002)	< 0.001	− 0.021 (0.003)	< 0.001	− 0.015 (0.002)	< 0.001	− 0.010 (0.003)	< 0.001
**Ethnicity (%)** **White dropped as baseline**								
**Mixed**	0.002 (0.005)	0.759	0.001 (0.008)	0.92	0.001 (0.005)	0.92	0.009 (0.008)	0.25
**Asian**	−0.012 (0.001)	< 0.001	−0.009 (0.002)	< 0.001	− 0.012 (0.001)	< 0.001	0.010 (0.002)	< 0.001
**Black**	−0.012 (0.003)	< 0.001	−0.026 (0.004)	< 0.001	− 0.015 (0.003)	< 0.001	− 0.023 (0.005)	< 0.001
**Other**	− 0.020 (0.008)	0.017	− 0.020 (0.012)	0.11	− 0.021 (0.008)	0.01	0.018 (0.012)	0.14
**Religious denomination (base = no religion)**								
**Catholic**	−0.046 (0.063)	0.463	0.210 (0.091)	0.02	−0.005 (0.062)	0.94	0.450 (0.008)	< 0.001
**Church of England**	−0.051 (0.037)	0.172	0.053 (0.054)	0.32	−0.020 (0.037)	0.58	0.165 (0.053)	< 0.001
**Other Christian Faith**	0.276 (0.456)	0.545	−0.105 (0.658)	0.87	0.104 (0.445)	0.81	−0.351 (0.660)	0.59
**Sikh**	0.486 (0.481)	0.312	−0.194 (0.682)	0.78	0.407 (0.471)	0.39	−1.279 (0.751)	0.09
**Other**	0.798 (0.463)	0.085	0.266 (0.672)	0.69	0.833(0.454)	0.07	−1.084 (0.655)	0.10
**High deprivation, quintile = 1** **(base = quintiles 2–5)**	−0.073 (0.054)	0.171	−0.165 (0.077)	0.03	−0.103 (0.053)	0.05	−0.193 (0.078)	0.01
**Urban** **(base = rural)**	0.062 (0.039)	0.115	0.004 (0.056)	0.94	0.055 (0.039)	0.15	−0.030 (0.056)	0.59
**Constant**	0.804 (0.049)	< 0.001	1.528 (0.07)	< 0.001	0.945 (0.048)	< 0.001	−2.506 (0.071)	< 0.001
**Variation between schools**	0.165 (0.010)	< 0.001	0.379 (0.023)	< 0.001	0.156 (0.010)	< 0.001	0.308 (0.021)	< 0.001
**Variation between school year nested in schools**	0.042 (0.005)	< 0.001	0.066 (0.007)	< 0.001	0.043 (0.005)	< 0.001	0.047 (0.010)	< 0.001
**ICC**	0.047 (0.003)		0.102 (0.005)		0.045 (0.003)		0.085 (0.005)	
**Wald chi2(3)**	399.18		335.62		448.13		169.83	
**Prob > chi2**	< 0.001		< 0.001		< 0.001		< 0.001	
**LR test**	3356.94		5122.97		2996.16		1811.57	
**Prob > LR**	< 0.001		< 0.001		< 0.001		< 0.001	
***n***	3010		2994		2994		2994	

^a^For 16 schools in the trial, an inconsistency in the data for number of consent forms returned led us to drop these observations from analysis of return of consent forms (Models 2–4), although they were included in the analysis of the primary outcome measure (uptake, Model 1)

Of the demographic factors, the percentage of pupils in the school who receive free school meals was negatively associated with uptake, *β* = − 0.017, *p* <  0.001, as was the percentage of students of Black ethnicity and percentage of Asian ethnicity, both of which had *β* = − 0.012, *p* <  0.001. There was no statistically significant effect of the school being a faith school associated with a particular religious denomination, being in the most deprived quintile by postcode, or being urban (versus rural). Variation remained between schools (var = 0.165, *p* <  0.001) and between school year nested in schools (var = 0.042, *p* <  0.001). The full model is shown in Model 1, Table 7.

The interventions increased the proportion of eligible children whose consent form was returned: In the control group, an average of 73.6% of eligible children in each school year returned their consent forms, compared to 75.7% in the letter group, 76.2% in the reminder group, and 76% in the letter and reminder group (see Table 6). When a multi-level model was run that included school and school year nested within school as random effects, which adjusted for demographic variables, there was a statistically significant effect of the letter (*β* = 0.129, *p* = 0.03) and of the reminder, (*β* = 0.150, *p* = 0.01), but no evidence of an effect of the interaction of letter and reminder (*β* = − 0.118, *p* = 0.16). See Model 2 in Table 7.

Receiving the reminder led to an increase in the proportion of eligible children for whom consent was granted, *β* = 0.100, *p* = 0.01; neither the letter nor the interaction of letter and reminder had statistically significant effects (see Model 3, Table 7). In the control group, an average of 64.9% children in each year had consent granted, compared to 65.5% in the letter only group, 67% in the reminder group and 66.3% in the letter and reminder group (see Table 6).

However, receiving the reminder led to an increase in the proportion of eligible children for whom consent was granted, *β* = 0.100, *p* = 0.01 neither the letter nor the interaction of letter and reminder had statistically significant effects (see Model 4, Table 7). In the control group, an average of 8.6% children in each year had consent refused, compared to 10.2% in the letter only group, 9.2% in the reminder group and 9.7% in the letter and reminder group (see Table 6).

Some schools had very small numbers of pupils and our results were robust to their exclusion from the analyses. We reran the model of our primary outcome variable (Model 1 in Table 7), excluding all year groups with less than ten pupils (144 datapoints). This did not have any effect on which variables had a statistical significance of *p* <  0.05.

## Discussion

We explored the effects of a behaviourally-informed letter on uptake of childhood influenza vaccination in general practices, and of a letter and a reminder (SMS/email) on uptake at schools. The behaviourally-informed, centrally sent invitation letter increased the proportion of children who received a vaccination at their general practice in absolute terms by 13.7% (from 23.4 to 37.1%). The effect of the invitation letter remained significant after adjusting for demographic variables and interaction effects, with children in the intervention group being more likely to be vaccinated than the control, OR = 2.17, *p* <  0.001, which is a medium-sized effect [31].

The effect of the interventions in schools was much smaller, though still statistically significant, with uptake ranging from 61.6% of the school year in the control group, who received the standard invitation letter and no reminder, to 63.5% in the group who received the standard letter and a reminder, an increase of almost 2% in absolute terms. In a fully adjusted model receiving a reminder had a statistically significant effect on vaccine uptake, *β* = 0.086, *p* = 0.036, but the behaviourally informed letter did not have a statistically significant effect.

Our behaviourally-informed letter increased uptake of the flu vaccine in general practice but it did not increase uptake in schools. In both studies, the control groups received usual practice. In the schools study, there was a standard letter invitation letter sent to all parents from the provider. In the general practice study, control practices and intervention practices may have sent parents an invitation letter, issued an opportunistic invitation, or not sent an invitation at all directly. GPs are contracted to call and recall for flu vaccinations. The National Institute of Health and Care Excellence (NICE) recommends contacting people for immunisations by a variety of methods, including text messages, letters, emails, phone calls from staff or an auto dialler, social media, or a combination of methods, as well as informing and inviting people during face-to-face interactions whenever the opportunity arises [30]. However, we do not know if the GP practices actually issued invitations; the comparison with the schools study suggests that they may not. It is important that they send letters because parents may expect an invitation for their child’s vaccinations; failing to receive one can be a reason for not attending [13]. Therefore, it may be the fact that our invitation was centrally-issued rather than the specific behavioural content that led to the increase in uptake; sending parents invitations is important.

There were some differences between the behavioural letter in the general practice and the schools study. The schools letter did not use personalisation, as that could not be implemented; nor was there was a tear off slip for an appointment, since the action was to complete and return the consent form. The schools letter had a positive social norms statement that was not in the GP letter. Since these differences between the letters are confounded with the difference in controls, we cannot be completely sure that the difference in effectiveness was due to the control (receipt of a letter invitation vs usual practice) or due to the differences in the letter (personalisation vs than norms).

Another difference between the GP and the schools trials is that the school-based programme sends a leaflet with the invitation letter, which has information about side-effects. In the behaviourally-informed letters, we did not address concerns about the vaccine’s effectiveness and side-effects, which have been shown to cause influenza vaccine hesitancy in parents [4, 9–13]. In other health domains, such as the NHS Health Check, letters that gave counter-arguments to common reasons for not attending have proved successful in increasing uptake [32]. However, a trial of behaviourally-informed patient information leaflets for the NHS Health Check leaflet did not find a significant effect, possibly because people do not pay attention to leaflets enclosed with invitation letters [33]. The effect of addressing concerns about vaccine hesitancy within the letter is a potential future avenue for research.

In an intention-to-treat analysis, our reminder led to a small increase in the proportion of children being vaccinated in schools, at the cost of an additional workload for schools. We collected data on fidelity to the study protocol in Dorset and the fidelity was poor: the providers did not necessarily use the wording that we gave them. We also found that a lot of the control schools were sending out reminders. We expect that the reminder would have increased uptake by increasing the return of consent forms; we found that both the letter and the reminder increased the number of consent forms returned (with no interaction effect), with increases in the proportion of forms returned of up to 2.6% in the reminder only condition compared to the control, taking the percentage for forms returned up to 76%. But there were still ~ 25% of parents who were not returning the consent forms in each condition, which suggests that there may be further scope for interventions that target the return of consent forms, especially given that 92.7% of people in the UK agree that vaccines are important for children to have [34]. Other techniques to improve uptake could include electronic consent, with parents consenting by SMS or email in reply to the reminder, or sending parents multiple reminders. However, the effect of sending one reminder was small and one might already ask questions about its cost effectiveness. Multiple reminders would further increase the workload for schools, with possibly diminishing returns.

Further, we found that the proportion of forms returned with consent refused increased when the intervention letter was sent, with increases of up to 1.6% in the letter only condition, taking refusals up to 10%. While reminders can be effective for parents who want their child to be vaccinated but do not get around to it [13], there is a ceiling effect on the effectiveness of reminders because we would not expect them to affect people who are vaccine hesitant. In the UK, 85.4% agree that the seasonal influenza vaccine is safe and 80.7% that it is important [34]. Parents may also decline the vaccine for faith reasons, due to the presence of porcine gelatine in the vaccine [4]. Again, this could contribute to a ceiling effect on reminders; in the UK, 81.6% agree that vaccines are compatible with their religious belief [34]. For those groups who are vaccine hesitant, and would actively refuse to let their children be vaccinated, we will need to design interventions that target the causes of their vaccine hesitancy.

This could also be an explanation for why our GP trial had larger effects. The baseline uptake rate in schools is higher than in general practice, so the interventions in schools are more likely to have been subject to a ceiling effect. The difference in baseline uptake rate is not surprising. The two settings target children of different ages, with the school children being older than those vaccinated in general practice, and data suggests that vaccine uptake decreases as age increases [35]. Because the schools vaccine programme already has a higher uptake, it may already have reached many of the parents who would accept a vaccine for their children. If many of the remaining parents are vaccine hesitant, then an approach that specifically targets the causes of their vaccine hesitancy may be needed to increase uptake.

Across all groups in the school study, we found that uptake of the vaccination decreased with the percentage of children in the school who were eligible for school meals, *β* = − 0.017, *p* <  0.001, and with the percentage of Black and Asian students, both *β* = − 0.012, *p* <  0.001. This is consistent with prior analyses, which have shown that there is lower uptake for children in higher areas of deprivation [3] and children of non-White ethnicity [7]. However, we do not know whether there were differences in the effectiveness of the intervention across demographic groups.

In the general practice study, children who had been vaccinated the previous year were more likely to be vaccinated, OR = 3.83, *p* <  0.001 a medium-large effect [31]. This could only apply to 3-year olds, as 2-year olds would have been too young to be eligible the previous year. However, it is not just an effect of age, since the model controlled for the age of the child, which was not statistically significant. There was no differential effect of the intervention across those who had and had not received a vaccination the previous year. Practices who used SystmOne had higher uptake. This could reflect a difference in practices who choose to use the SystmOne software or be due to SystmOne itself, which extracts immunization data automatically, rather than relying on manual input.

In the general practice study, we found variation between practices and CCGs, and in the school study we found variation between both school years and schools, suggesting that there are practice/school level factors that influence uptake. The CCG-level differences are consistent with other data, which shows that uptake of the flu vaccine for 2- to 3-year olds varies from 19 to 61% across CCGs [7]. In the case of the GP practices, there is no evidence of difference in the amount of practice variation between the intervention and control groups. In the case of the schools, some of the inter-school variation was probably accounted for by the differing demographics of the schools, since the variation decreased when we put demographic factors into the model. However, it remained statistically significant even after the addition of demographic control variables.

## Limitations

Process evaluations for each study, which would have enhanced our contextual understanding of the study outcomes, were not conducted. Future research investigating the effectiveness of behaviourally-informed letters should therefore undertake process evaluation to better understand how and why the interventions may be effective. Also, the trials were not designed to be compared. As such there are many differences between the two trials (i.e., setting, age group, and trial design) which make comparing the results from each trial to better understand the determinants of an effective letter intervention difficult.

## Conclusion

Sending a behaviourally-informed invitation letter can increase uptake of childhood influenza vaccines at the GP surgery compared to usual practice. A reminder SMS or email, which follows on from an invitation letter, can lead to a small increase in uptake of the influenza vaccine in schools.

## Supplementary Information

**Additional file 1: Supplementary File 1.** GP Flu Vaccine Invitation Letter. This file contains the vaccine invitation letter sent out by the GP to patients.

**Additional file 2: Supplementary File 2.** School Flu Behavioural Invitation Letter. The file contains the behaviourally-informed letter which was sent by providers of childhood flu school vaccination to parents at those schools.

## Data Availability

The datasets generated and analysed during the current study are available from the corresponding author on reasonable request.

## Abbreviations

GP: General Practices
BAME: Black and Ethnic Minority
NCMP: National Child Measurement Programme
BMI: Body Mass Index
RTI: Randomised Controlled Trial
PHEBI: PHE Behavioural Insights
CCG’s: Clinical Commissioning Groups
BCT: Behavioural Change Technique
CHIS: Child Health Information System
NICE: National Institute of Health and Care Excellence
IMD: Index of Multiple Deprivation
GLM: Generalised Linear Model

## Footnotes

1 https://www.gov.uk/government/collections/annual-flu-programme

2 Available at https://www.compare-school-performance.service.gov.uk/download-data
