# Supplementary material for: Can uptake of childhood influenza immunisation through schools and GP practices be increased through behaviourally-informed invitation letters and reminders: two pragmatic randomized controlled trials
Source: BMC Public Health. 2023 Jan 20;23:143. doi: 10.1186/s12889-022-14439-4 (PMC9854224; doi:10.1186/s12889-022-14439-4)
Supplement: Supplementary file 1 — Additional file 1: Supplementary File 1. GP Flu Vaccine Invitation Letter. This file contains the vaccine invitation letter sent out by the GP to patients. [file 12889_2022_14439_MOESM1_ESM.pdf]

Supplementary File 1: GP Flu Vaccine Invitation Letter
Description: This file contains the vaccine invitation letter sent out by the GP to patients.

[GP Surgery] T [000 000 0000]
[First address line]
[Second address line]
[Town/city]
[County Postcode]

[Date]

Dear [Name]

«**Insert child's first name**»**'s annual flu vaccination is now due.**

This vaccination programme is in place to help protect your child against flu. Flu can be an unpleasant illness and sometimes cause serious complications. Vaccinating your child will also help protect more vulnerable friends and family by preventing the spread of flu.

Please phone [insert number] to book an appointment for [insert child's name]'s flu vaccination.

The vaccination is free and recommended for young children, and will be given by a quick and simple spray up the nose.

We look forward to seeing «**Insert child's first name**» soon.

Yours sincerely,

[GP/Practice Nurse/Practice Manager name]

[Position/title]

For more information visit: www.nhs.uk/child-flu

--✂---

Please record the date and time of your child's vaccination appointment here and put it in an obvious place in your home.

Vaccination appointment:

on: _____/_____/_____ at _________am/pm

Date **Time**
