# Supplementary material for: Can uptake of childhood influenza immunisation through schools and GP practices be increased through behaviourally-informed invitation letters and reminders: two pragmatic randomized controlled trials
Source: BMC Public Health. 2023 Jan 20;23:143. doi: 10.1186/s12889-022-14439-4 (PMC9854224; doi:10.1186/s12889-022-14439-4)
Supplement: Supplementary file 2 — Additional file 2: Supplementary File 2. School Flu Behavioural Invitation Letter. The file contains the behaviourally-informed letter which was sent by providers of childhood flu school vaccination to parents at those schools. [file 12889_2022_14439_MOESM2_ESM.pdf]

Supplementary File 2: School Flu Behavioural Invitation Letter
Description: The file contains the behaviourally-informed letter which was sent by providers of childhood flu school vaccination to parents at those schools.

*[Provider letterhead plus NHS logo]*

*TEMPLATE LETTER FOR SCHOOL-AGED CHILDREN*

[Date]

Dear Parent/Guardian,

**Your child’s annual flu vaccination is now due**

This vaccination programme is in place to help protect your child against flu. Flu can be an unpleasant illness and sometimes causes serious complications. Vaccinating your child will also help protect more vulnerable friends and family by preventing the spread of flu.

**Please complete the enclosed consent form** (one for each child) and return to the school [by/ within] [**INSERT DATE or TIME FRAME**] to ensure your child receives their vaccination.

The vaccination is free and recommended for young children, and will be given by a quick and simple spray up the nose.

A leaflet explaining the vaccination programme is enclosed and includes details about the small number of children for whom the nasal vaccine is not appropriate.

Last year, most children offered the vaccine in schools had the immunisation.

If you have any queries please contact the healthcare team on [INSERT NUMBER].

Yours sincerely,

[Signed by Provider]

**If your child becomes wheezy or has their asthma medication increased after you return this form, please contact the healthcare team on [phone number].**

If you decide you do not want to vaccinate your child against flu, please return the consent form giving the reason. This will help us plan and improve the service.

**For further information see: www.nhs.uk/child-flu**
